# A brief review of forensically important flesh flies (Diptera: Sarcophagidae)

**DOI:** 10.1080/20961790.2018.1432099

**Published:** 2018-03-22

**Authors:** Lipin Ren, Yanjie Shang, Wei Chen, Fanming Meng, Jifeng Cai, Guanghui Zhu, Lushi Chen, Yong Wang, Jianqiang Deng, Yadong Guo

**Affiliations:** a Department of Forensic Science, School of Basic Medical Sciences, Central South University, Changsha, China; b Department of Forensic Medicine, Shantou University Medical College, Shantou, China; c Department of Forensic Medicine, Guizhou Police Officer Vocational College, Guiyang, China; d Department of Forensic Medicine, Hainan Medical University, Haikou, China

**Keywords:** Forensic science, forensic entomology, sarcophagid flies, decomposition, biology, distribution

## Abstract

Forensic entomology could provide valuable data for the minimum postmortem interval (PMI_min_) estimation and other relevant information, such as causes and circumstances of death. Some representatives of flesh flies are one of the dominant necrophagous insects during early stages of decomposition, demonstrating unique biological characteristics compared with other necrophagous flies. Moreover, they lead to global health concerns as carriers of various pathogenic micro-organisms, and dominantly result in the traumatic myiasis. Thus, sarcophagid flies are considered important in decomposition processes for PMI_min_ estimation. However, the utility of sarcophagid flies has been seriously hampered by limited ecological, biological and taxonomic knowledge of them. The aim of this paper is to provide a brief review on the species, distribution and biological habit of forensically important sarcophagid flies. In addition, the relation between traumatic myiasis and flesh flies, molecular identification methods and developmental pattern of flesh flies are summarized.

## Introduction

The correct sampling, measuring and subsequent interpretation of the insects found on decomposed remains would provide valuable information in forensic science, such as the minimum postmortem interval (PMI_min_), the causes and circumstances of the death, toxication and human DNA from the gut of the larvae [1,2]. By determining the developmental stage of necrophagous insects colonized on decomposed remains and the initial colonization timeframes, the PMI_min_ estimation for decomposed corpses is relatively accurate [3]. The common necrophagous insects are Diptera order, mainly including Sarcophagidae, Calliphoridae and Muscidae family, which are critically important in forensic investigations [4–6].

Sarcophagid flies (known as flesh flies) visiting a corpse mostly belong to the synanthropic dement of subtropical or even tropical origin, which constitute a part of the insect faunal succession representing actually the first and very important destruction stage responsible for the essential decomposition [7–9]. Nevertheless, compared with other fly species, sarcophagids have unique characteristics facilitating the estimation of PMI_min_. First, many flesh flies are well known for adopting the reproductive strategy of ovoviviparity (or ovolarviparity); they deposit maggots directly on a corpse instead of eggs [4,9,10]. Second, they are more observable than others because of the larger size [5,7,11]. Third, sarcophagid flies are more active in various decay stages of corpses [6,8,12]. Moreover, they may play important role in decomposition of buried carrion since they are more efficient colonizers for these types of substrates than blowflies [13,14].

As mentioned above, sarcophagid flies should be widely applied to estimate the PMI, whereas in forensic investigations, it is severely limited by the insufficiency of systematic studies on the taxonomic features and inadequate documentation of their thermobiological histories. Establishment of detailed database on the flesh flies is vitally important. Hence, the aim of this review is to provide a comprehensive review on the species and distribution of sarcophagid species in forensic investigations, especially in indoor cases. Besides, reports of traumatic myiasis caused by sarcophagid species, the effect of drugs on the growth rates of flesh flies, species identification and the developmental pattern of flesh flies are summarized.

## Species diversity and distribution of flesh flies

Sarcophagid flies distribute worldwide, and consist of more than 100 genera and 2600 species, among which approximately 800 species belong to the genus Sarcophaga [5,7,8,11,15,16]. Since the dominant species vary significantly with geographic region and climate [10], insect faunal succession on decaying carcasses concerning flesh flies were currently performed, e.g. in Finland, Switzerland, Portugal, Germany, Poland, Spain, Italy, Brazil, United States, India, Australia, Malaysia, Thailand, Japan, Egypt and China.

The geographic region or biogeoclimatic zone has a major impact on the species of insects existed on a corpse. For instance, *Sarcophaga africa* (Wiedemann), *Sarcophaga argyrostoma* (Robineau-Desvoidy), *Sarcophaga caerulescens* Zetterstedt, *Sarcophaga dux* Thomson, *Sarcophaga melanura* Meigen and *Sarcophaga similis* Meade are dominant species in Europe (e.g. Finland, Switzerland, Germany, Spain and Poland) [10,17–20]. The species of *Sarcophaga peregrina* (Robineau-Desvoidy), *Sarcophaga ruficornis* (Fabricius) and *Sarcophaga taenionota* Wiedemann are widely distributed in China and Malaysia [12,21–26]. *Sarcophaga albiceps* Meigen is extensively found in Asia (e.g. China, India and Malaysia) [9,12,22,26], and Europe (e.g. Germany and Poland) [10,17]. *Sarcophaga crassipalpis* Macquart is widespread in Spain, Australia and China. *Wohlfahrtia nuba* (Wiedemann) is frequently recorded in the Middle East (e.g. Egypt and Kuwai) [27,28]. Moreover, a new record of *Sarcophaga cultellata* Pandelle was identified at preimaginal stages collected in autopsies performed in Spain, which is reported for the first time in human corpses [29]. The detailed summary is shown in Table 1.

**Table 1.** The common species and distribution of forensically important flesh flies.

No	Species	Location	Animal model	Habitat	Date of collection	References
1	*Boettcherisca highlandica* Kurahashi & Tan	Malaysia (Pahang)	Rabbits	Highland	Unstated	[12]
2	*Blaesoxipha plinthopyga* (Wiedemann)	USA (Idaho)	Human	Mountain	August 2002	[30]
3	*Liosarcophaga babiyari* (Lehrer)	Saudi Arabia (Al-Baha)	Rabbits	Mountain	Unstated	[31]
4	*Oxysarcodexia intona* (Curran & Walley)	Brazil (Maranhão)	Baited traps	Outdoor	2009–2012	[32]
		Brazil (Recife)	Pigs	Rainforest	Unstated	[33]
5	*Oxysarcodexia riograndensis* Lopes	Brazil (Pernambuco)	Human	Rural	2008	[34]
		Brazil (Recife)	Pigs	Rainforest	Unstated	[33]
6	*Oxysarcodexia thornax* (Walker)	Brazil (Maranhão)	Baited traps	Outdoor	2009–2012	[32]
		Brazil (São Paulo)	Baited traps	Rural, urban, forest	September 2009–August 2010	[35]
7	Peckia chrysostoma* (Wiedemann)	Brazil (Pernambuco)	Male cadaver	Indoor	July 2012	[36]
		Brazil (Maranhão)	Baited traps	Outdoor	2009–2012	[32]
8	*Peckia* (*Squamatodes*) *ingens* (Walker)	Brazil	Baited traps	Outdoor	2009–2012	[32]
			Baited traps	Outdoor	Unstated	[34]
			Pig	Rainforest	Unstated	[33]
9	*Peckia* (*Sarcodexia*) *lambens* (Wiedemann)	Brazil	Baited traps	Outdoor	2009–2012	[32]
			Baited traps	Outdoor	Unstated	[34]
			Baited traps	Rural, urban, forest	September 2009–August 2010	[35]
10	*Ravinia belforti* (Prado & Fonseca)	Brazil (Pernambuco)	Human corpse	Rural	2008	[34]
11	*Ravinia pernix* (Harris)	Saudi Arabia (Riyadh)	Rabbits	Agricultural/desert/urban area	June 2014	[37]
12	Sarcophaga aegyptiaca*Salem	Egypt (El-Qalyubiya)	Rabbit	House	August–September 2008	[27]
13	*Sarcophaga albiceps* Meigen	China (Zhongshan)	Pigs	Outdoor	December 2003–October 2004	[22]
		China (Guizhou)	Pigs	Outdoor	April 1998–April 1999	[26]
		India (Punjab)	Mutton	Wooden platform	September 2005	[9]
		Germany (Frankfurt)	Baited traps	Rural	September 2008–May 2011	[17]
		Malaysia (Pahang)	Rabbits	Highland	2011–2012	[12]
		Poland	Pig	Forest and grassland	Unstated	[10]
		India (Punjab)	Rabbits	Campus area	March 1997–December 1999	[38]
14	Sarcophaga africa*(Wiedemann)	Switzerland (canton de Vaud)	Human	Indoor	Unstated	[19]
		Spain (Alcala´ de Henares)	Carrion-baited traps	Urban	October 2005–September 2006	[20]
		Poland	Pig	Forest and grassland	Unstated	[10]
		Kuwait	Rabbits	Outdoor	2009	[28]
15	Sarcophaga argyrostoma* (Robineau-Desvoidy)	Switzerland (canton de Vaud)	Human	Indoor	Unstated	[19]
		Poland	Pig	Forest and grassland	Unstated	[10]
		Spain (Alcala´ de Henares)	Carrion-baited traps	Urban	Unstated	[20]
		Central Europe	Woman	Indoor	April 1993	[14]
16	Sarcophaga caerulescens* Zetterstedt	Southern Finland (Turku)	Human	Indoor	Unstated	[39]
		Switzerland (canton de Vaud)			Unstated	[19]
		Germany (Frankfurt)	Baited traps	Rural	September 2008–May 2011	[17]
		Poland (Biedrusko)	Pigs	Grassland	2012–2014	[18]
		Poland			Unstated	[10]
17	*Sarcophaga carnaria* (Linnaeus)	Germany (Frankfurt)	Baited traps	Rural	September 2008–May 2011	[17]
18	Sarcophaga crassipalpis* Macquart	Spain (Alcala´ de Henares)	Carrion-baited traps	Indoor	October 2005–September 2006	[20]
		Australia (Queensland)	Human	Indoor	December 2011–January 2014	[24]
		China (Shenzhen)	Man, pig and rabbit	Forest	August 2013	[21]
		Japan (Saitama)	Human	Unstated	July–September/Unstated	[40]
		Moravia (Brno)	Woman	Indoor	August 1992	[8]
19	*Sarcophaga cultellata* Pandelle	Spain	Human corpse	Unstated	Unstated	[29]
20	*Sarcophaga dux* Thomson	Japan (Saitama)	Human	Unstated	July–September/Unstated	[40]
		Switzerland (canton de Vaud)	Human	Outdoor	Unstated	[19]
		northern Thailand (Chiang Mai)	Baited traps	Outdoor	July 2002–February 2003	[41]
21	*Sarcophaga hirtipes* Wiedemann	India (Punjab)	Mutton	Wooden platform	September 2005	[9]
		India (Punjab)	Rabbits	Campus area	March 1997–December 1999	[38]
		Saudi Arabia (Riyadh)	Rabbits	Agricultural/desert/urban area	June 2014	[37]
22	Sarcophaga impatiens*Walker	Australia (Queensland)	Human	Indoor	December 2011–January 2014	[24]
23	*Sarcophaga melanura* Meigen	Poland	Pig	Forest and grassland	Unstated	[10]
		Spain (Alcala´ de Henares)	Carrion-baited traps	Periurban	October 2005–September 2006	[20]
24	Sarcophaga peregrina*(Robineau-Desvoidy)	China	Pigs	Indoor	April 1998–April 1999	[26]
			Human	River	July 2010	[23]
			Man, pig and rabbit	Forest	August 2013	[21]
		Malaysia (Terengganu)	Rabbits	Rural	2011–2012	[12]
		Japan (Saitama)	Human	Unstated	July–September/Unstated	[40]
		Northern Thailand (Chiang Mai)	Baited traps	Outdoor	July 2002–February 2003	[41]
25	*Sarcophaga praedatrix* Walker	Australia (Queensland)	Human	Grassland	2011–2012	[24]
26	*Sarcophaga princeps* Wiedemann	Malaysia	Human, Rabbits	Outdoor	July 2007–July 2010	[25]
					Unstated	[11]
		India (Punjab)	Rabbits	Campus area	March 1997–December 1999	[38]
27	Sarcophaga ruficornis*(Fabricius)	Australia (Queensland)	Human	Indoor	December 2011–January 2014	[24]
		Malaysia (Penang)			July 2007–July 2010	[25]
		China (Zhongshan)	Pigs	Outdoor	December 2003–October 2004	[22]
		Kuwait	Rabbits	Outdoor	2009	[28]
		northern Thailand (Chiang Mai)	Baited traps	Outdoor	July 2002–February 2003	[41]
28	Sarcophaga similis*Meade	Switzerland (canton de Vaud)	Human	Indoor	Unstated	[19]
		Poland (Biedrusko)	Pigs	Grassland	Unstated	[34]
		Poland	Pigs	Forest and grassland	Unstated	[10]
		Germany (Frankfurt)	Baited traps	Urban	September 2008–May 2011	[17]
29	*Sarcophaga subvicina* Baranov	Germany (Frankfurt)	Baited traps	Rural	September 2008–May 2011	[17]
30	*Sarcophaga taenionota* Wiedemann	China (Zhongshan)	Pigs	Outdoor	December 2003–October 2004	[22]
		Malaysia (Pahang)	Rabbits	Rural/highland	Unstated	[12]
31	Sarcophaga tibialis*Macquart	Spain (Alcala´ de Henares)	Carrion-baited traps	Indoor	October 2005–September 2006	[20]
32	*Sarcophagavariegata* (Scopoli)	Germany (Frankfurt)	Baited traps	Rural	September 2008–May 2011	[17]
33	*Sarcophaga spp*.	Malaysia	Human	Indoor	2004	[42]
				Indoor	July 2007–July 2010	[25]
				Indoor (high-rise buildings)	2015	[43]
				Indoor	January 2010–December 2013	[44]
		Italy (Tuscany)		Indoor	2009–2010	[45]
34	*Tricharaea occidua* (Fabricius)	Brazil (Maranhão)	Baited traps	Outdoor	2009–2012	[32]
35	*Wohlfahrtia nuba* (Wiedemann)	Kuwait	Rabbits	Outdoor	2009	[28]

*The common species and distribution of flesh flies with indoor activity habits.

The diversity and abundance of biases towards flesh flies may be explained by habitat preferences, as they are strongly synanthropic [10,17]. Fremdt and Amendt [17] demonstrated that *Sarcophaga subvicina* Baranov, and *Sarcophaga variegata* (Scopoli) could serve as indicators of urban habitats during summer and *S. albiceps* as indicator of rural habitats in Frankfurt, Germany. A significant association of *S. caerulescens* with rural habitats as well as *S. similis* with urban habitats was observed [17]. Geographical region has obvious influence on arrival time of different species of insects, suggesting that data generated in one region or biogeoclimatic zone cannot be used as a direct reference to estimate the PMI in a different region. It is recommended that databases should be developed for every biogeoclimatic zone in which insects are used to estimate the time of colonization.

## Effect of indoor environment on flesh flies

Flesh flies were widely reported to colonize on indoor corpses, which may be due to the special biological features [30,46–48]. In recent years, flesh flies were frequently found to invade corpses in indoor cases, which were mainly reported in Japan, Southern Finland, Switzerland, Spain, Australia, Brazil, United States, Malaysia, Italy, Poland and China. In Switzerland, *S. caerulescens*, *S. similis* and *S. africa* have been reported to be the dominant species colonizing on the corpses in indoor cases, and *S. argyrostoma* was commonly found indoors during summer [19]. Meanwhile, the involvement of *S. argyrostoma* in indoor cases has also been reported in Poland [49]. In Italy, *S. africa* was also recorded in indoor cases [45]. However, it should be treated with caution when estimating the PMI_min_ according to the developmental data of the larvae of *S. africa* on human corpses, as it is well known that this fly prefers to larviposit of faeces [50]. Moreover, *S. caerulescens* was dominant species found in indoor corpses in Finland [39]. In conclusion, *S. peregrina*, *S. ruficornis* and *S*. (*Liosarcophaga*) *tibialis* Macquart were often reported in China, Spain and Australia, respectively [20,24,26]. *Sarcophaga crassipalpis* and *Sarcophaga impatiens* Walker were also found to colonize on the corpses at the earliest stage of decomposition in Australia [24].

Additionally, Syamsa et al. [43] reported the occurrence of flesh flies at higher altitudes. Unfortunately, the authors failed to identify them to the species level because of insufficient taxonomical studies regarding the larvae of this taxon. In summary, more than 10 common species of flesh flies typically colonize on indoor cadavers, including *S. africa*, *S. argyrostoma*, *S. caerulescens*, *S. crassipalpis*, *S. peregrina*, *S. ruficornis*, *S. similis*, etc. (Table 1). Even so, the insufficient taxonomic and developmental data of flesh flies severely limit their application in the PMI estimation compared with blowflies.

## Influence of drugs on flesh flies

Certain cases of drug-related deaths occurred in concealed places, particularly for solitary victims. The cadavers are usually found at the later stages of decomposition. Although it is difficult to estimate the PMI according to the postmortem phenomena, forensic entomology has unique advantages in such cases [51–59], whereas, if the effects of drugs on the developmental pattern of flies are not taken into account, misestimate of PMI might occur. Therefore, knowledge of various drugs on the development of immature carrion-breeding insects could be potentially valuable in redefining the PMI estimation, which involves deducing minimum and maximum PMI [60].

Drugs can affect the developmental pattern of flesh flies, potentially leading to the misestimation of PMI. As early as 1989–1991, Goff et al. [55,56] reported that cocaine and heroin residues and metabolites accelerated the development of the larvae of *S. peregrina*. Later, Goff et al. [57,61] reported again that higher concentrations of methamphetamine (‘ice’) accelerated the development of *S. ruficornis*, and lower concentrations of 3, 4-methylenedioxymethamphetamine (MDMA) delayed the larval development of the same species. Whereas, puparial durations of *S. ruficornis* were significantly longer for the colonizers fed on tissues from the rabbits receiving the high concentrations of amitriptyline and phencyclidine [58,59]. These effects could potentially lengthen the PMI estimation up to 70 h [58]. In South Africa, Musvasva et al. [53] demonstrated that the larvae of *S. tibialis* exposed to the hydrocortisone and sodium methohexital took significantly longer time to reach pupation compared with those in the control while the larvae exposed to sodium methohexital passed through pupation significantly faster than those in the control. Yet, no systematic relationship was found between drug concentration and developmental time of larvae or pupae. The total developmental period from hatching to eclosion did not differ after drug treatments, implying that estimation of the PMI based on the emergence of adult flies will not be affected by the involvement of these drugs in a case. On the other hand, anomalous pupation spans might indicate the presence of barbiturates. Recently in China, Zhang et al. [62] explored that the larvae of *S. crassipalpis* grew faster with the increased concentration of morphine hydrochloride. Moreover, Goff et al. [55,56,58] also emphasized the need for studies on the effects of more drugs on the development of various species of necrophagous flies. Thus, further analyses involving different fly species, drug types, concentrations and means of administration should be undertaken to establish a systematic database in support of criminal investigations.

Besides, sarcophagids and their remains could be used for entomological toxicology (entomotoxicology) analyses. Entomotoxicology is the science studies the potential use of insects for detecting drugs or other toxic substances that may not be measurable in decomposing tissues. Necrophagous insects, feeding on the decomposing remains, accumulate toxins present in their food substrates. These insects, in some cases, provide a more reliable and sensitive result than traditional analytical methods dealing with decomposed tissues [52].

## Relation between traumatic myiasis and flesh flies

Myiasis is the invasion of tissues and organs both in humans and animals by dint of the larvae of sarcosaprophagous flies. Those larvae feed on the host tissues, body fluids, or ingested food as parasites in the skin, subcutaneous tissues, mouth, stomach, eyes, nose, ears, intestines, urinogenital system, and other soft tissues of humans and warm-blooded vertebrate animals [63]. Relevant cases were mainly reported in Europe and Asia at present. In humans and animals, sarcophagid species have been reported to cause myiasis in ophthalmic, nasal, urinogenital, aural, cutaneous, oral and gastrointestinal cases [64–89]. Accordingly, it is crucial to exclude traumatic myiasis in the PMI estimation based on the development of sarcosaphagous flies [63]. Investigations illustrated that the most common species causing traumatic myiasis is *Wohlfahrtia magnifica* Schiner, Wohlfahrt's wound myiasis fly, the third of the most important obligatory traumatic myiasis agents [63,90]. Besides, the common sarcophagid species causing myiasis also includes *S. africa*, *S. argyrostoma*, *S. crassipalpis* and *S. ruficornis*.

Traumatic myiasis caused by sarcophagid species is extensively reported as the consequence of ignorance and can be used as an indicator of wound care neglect, either by oneself or by the nurses [63]. Obviously, criminal investigations require more researches involving various fly species and means of administration to establish a systematic database.

## Species identification of flesh flies

Although the species of sarcophagids can be identified by their morphological characteristics of male terminalia, they present as being very numerous and diverse [10,91,92]. Thus, species identification based on morphological methods requires specialized taxonomic knowledge, only a few specialists are able to identify larvae of forensically relevant insects to species level [13,93]. To implement the use of sarcophagids for PMI estimation, a method for easy and accurate species-level identification at any life stage is required. DNA-based method is an alternative method proposed to identify species credibly and rapidly with lower requirement of sample preservation. DNA sequence data would serve as standards for further analysis [94]. Phylogenies also improve the understanding of the taxonomy and systematics of flesh flies [95–99].

At present, the partial genes of mitochondrial genome have been broadly applied to the species-level identification, mainly including the different fragments of *Cytochrome c oxidase subunit I* (*COI*) gene [94,95,100–115], in addition to the *Cytochrome c oxidase subunit II* (*COII*) gene [108–113], 16S ribosomal RNA (16S rRNA) [108–119], 12S ribosomal RNA (12S rRNA) [119], the nicotinamide adenine dinucleotide (NADH) dehydrogenase subunit 5 [108,109], the ribosomal internal transcribed spacer regions [119,120] and the nuclear period and 28S rRNA genes [111,112] (Table 2). Although these markers could be potentially served as discriminatory tools in identification of forensically important flesh flies, available gene sequences are deficient in the species-level identification of Sarcophagidae on GenBank databases, such as a flaw of insufficient discrimination power in utility of short gene fragments. The use of complete gene remains time-consuming and has a higher requirement for the preservation quality of specimens [104]. Until recently, a set of 4-SNP marker system has been developed for the identification of forensically important sarcophagid flies using the Pyrosequencing (PSQ) method, which showed high discriminating power, specificity of PCR amplification and particular advantages for degraded insect samples [121].

**Table 2.** DNA-based identification of forensically important Sarcophagid flies.

No	DNA region	Amplified fragment length (bp)	Primer ID and sequences	Collection location	References
1	*COI*	783	Unstated	USA	[94]
2	*COI*	278	C1-J-2495: 5′-CAGCTACTTTATGAGCTTTAGG-3′ C1-N-2800: 5′-CATTTCAAGCTGTGTAAGCATC-3′	Australia	[95]
3	*COI*	304	5′-CTGCTACTTTATGAGCTTTAGG-3′ 5′-GATGCTTACACAACTTGAAATG-3′	Japan	[108]
4	*COI*	658	5′-GGTCWACWAATCATAAAGATATTGG-3′ 5′-RAAACTTCWGGRTGWCCAAARAATCA-3′	Australia	[101]
					[102]
5	*COI*	304	5′-CAGCTACTTTATGAGCTTTAGG-3′ 5′-CATTTCAAGCTGTGTAAGCATC-3′	China and Egypt	[103]
6	*COI*	127/658	TY-J-1460: TACAATTTATCGCCTAAACTTCAGCC C1-N-2191: CCCGGTAAAATTAAAATATAAACTTC C1-J-2183: CAACATTTATTTTGATTTTTTGG TL2-N-3014: TCCAATGCACTAATCTGCCATATTA 5′-AAAATTATAATAAARGCRTGRGC-3′ 5′-TCYACTAATCATAAAGATATTGGYAC-3′	West Europe	[104]
7	*COI*	272/1 173	272- COI: 5′-CAGATCGAAATTTAAATACTTC-3′ 5′-GTATCAACATCTATTCCTAC-3′ 1173- COI: 5′-TACAATTTATCGCCTAAACTTCAGCC-3′ 5′-CAGCTACTTTATGAGCTTTAGG-3′	Egypt and China	[105]
8	*COI*	465	5′-CAGCTACTTTATGATCTTTAGG-3′ 5′-CATTTCAAGCTGTGTAAGCATC-3′	India	[106]
9	*COI*	400	Unstated	Brazil	[107]
10	*COI* + *ND5*	296 + 386	COI: 5′-CAGCTACTTTATGATCTTTAGG-3′ COI: 5′-CATTTCAAGCTGTGTAAGCATC-3′ ND5: 5′-CCAAAATATTCTGATCATCCTTG-3′ ND5: 5′-GGATTAACTGTTTGTTATACTTTTCG-3′	Germany	[108]
				India	[109]
11	*COI* + 16SrDNA	278 + 289	C1-J-2495: 5′-CAGCTACTTTATGAGCTTTAGG-3′ C1-N-2800: 5′-CATTTCAAGCTGTGTAAGCATC-3′ 5′-CGCTGTTATCCCTAAGGTAA-3′ 5′-CTGGTATGAAAGGTTTGACG-3′	China	[110]
12	*COI* + period	700 + 678	COI: 5′-CTTTACCTGTACTTGCTGGAG-3′ COI: 5′-AACTTGTCGTTGTGATGCT-3′ Period: 5′-CGCTGTTATCCCTAAGGTAA-3′ Period: 5′-CTGGTATGAAAGGTTTGACG-3′	China	[111]
13	*COI* + 28SrDNA	Unstated	Unstated	Thailand (Chiang Mai)	[112]
14	*COII*	189	5′-ATTAGATGTTGATAATCG-3′ 5′-ACAAATTTC-TGAACATTG-3′	China	[116]
15	*COII*	635	5′-AGAGCCTCTCCTTTAATAGAACA-3′ 5′-GAGACCATTACTTGCTTTCAGTCATC-3′	Egypt and China	[117]
16	*COII* + 16S rDNA	637 + 555	C2-J-3138: 5′-AGAGCCTCTCCTTTAATAGAACA-3′ TK-N-3775: 5′-GAGACCATTACTTGCTTTCAGTCATC-3′ LR-J-12 887: 5′-CCGGTCTGAACTCAGATCACGT-3′ LR-N-13 398: 5′-CGCCTGTTTAACAAAAACAT-3′	China	[118]
17	*COI* + *COII*	2 300	TY-J-1460: 5′-TACAATTTATCGCCTAAACTTCAGCC-3′ C1-N-2800: 5′-CATTTCAAGCTGTGTAAGCATC-3′ C1-J-2495: 5′-CAGCTACTTTATGAGCTTTAGG-3′ TK-N-3775: 5′-GAGACCATTACTTGCTTTCAGTCATCT-3′	Malaysia	[111]
				China	[114]
18	*COI* + *COII*	1 300	5′-CAGCTACTTTATGAGCTTTAGG-3′ 5′-GAGACCATTACTTGCTTTCAGTCATCT-3′	Egypt and China	[115]
19	12S and 16SrDNA + *ITS*	1 172 + 1 500	mtD-33F: 5′-ATGTTTTTGTTAAACAGGCG-3′ mtD-12SR: 5′-AAACTAGGATTAGATACCCTATTAT-3′ 18SF-1975F: 5′-TAACAAGGTTTCCGTAGGTG-3′ 28SR-52R: 5′-GTTAGTTTCTTTTCCTCCCCT-3′	Malaysia	[119]
20	*ITS2*	Unstated	ITS2_F: 5′-TGCTTGGACTACATATGGTTG A-3′ ITS2_R: 5′-GTAGTCCCATATGAGTTGAGGTT-3′	China	[120]
21	MtSNP markers	<150	Unstated	China	[121]

Due to the recent burst of development in forensic sciences, new court criteria require the evaluation of scientific evidence prior to its submission to the court [122]. Limitation of individual gene for species identification has been illustrated by recent studies [111,123]. Combined use of multiple genes is more valuable for evolutionary analysis and closely related species. To raise the identification efficiency of certain genes, the molecular markers still require further screening and optimization. Meanwhile, it is necessary to explore accurate, rapid and reliable species determination methods that are relatively insensitive to sample preservation so as to improve the application of flesh flies in forensic investigations.

## Developmental pattern of flesh flies

Generally, the developmental pattern of flesh flies is in a predictable manner under controlled temperature [93]. To ensure accurate PMI_min_ estimation, it is particularly important to collect precise basic data on the developmental pattern of flesh flies [124]. In 1994, Amoudi et al. [125] explored that the developmental time of *S. ruficornis* at constant temperatures varying from 13 °C to 37 °C, indicating that the optimal temperature in terms of rapid development, low mortality and greatest weight was from 22 °C to 28 °C. In 1998, Byrd and Butler [126] reported that the developmental durations from first instar to adult for the larva and pupa of *S. haemorrhoidalis* (Fallen) ranged from 252 h to 802 h under cyclic temperatures with means of 15.6 °C, 21.1 °C, 26.7 °C and 35 °C, and a constant temperature of 25 °C. In 2002, Grassberger and Reiter [127] studied the total developmental time of *S. argyrostoma* from larviposition to adult emergence was from (54.9 ± 1.45) to (14.9 ± 0.4) days reared at six constant temperature regimes (8 °C–35 °C), respectively. Moreover, the minimum development threshold for total immature development is 7.4 °C. In 2014, Mariana et al. [128] explored the rates of development, viability and survival of immature *S. ruficornis* and *Microcerella halli* (Engel) that were reared at different temperatures, demonstrating that the range of optimum temperature for *S. ruficornis* was between 20 °C and 35 °C, and that for *M. halli* was between 20 °C and 25 °C. Furthermore, for both species, the longest time of developmental duration was at the lowest temperature, and the survival rate was lower at extreme temperatures (10 °C and 35 °C). In 2017, Wang et al. [129] reported that the developmental durations of *S. peregrina* at seven constant temperatures (16 °C–34 °C) ranged from (1 064.7 ± 34.8) to (258.0 ± 3.5) h. Moreover, the developmental threshold temperature of *S. peregrina* was (10.87 ± 0.49)  °C, and the thermal summation constant was (5 809.7 ± 291.4) degree days. In the same year, Yang et al. [130] investigated the development patterns of *S. similis* which was reared at nine constant temperatures ranging from 15 °C to 35 °C (Table 3).

**Table 3.** The developmental pattern of forensically important flesh flies

No	Species	Temperature (°C)	First-instar (h)	Second-instar (h)	Third-instar (h)	Pupa (h)	Total duration (h)	References
1	*Microcerella halli* (Engel)	10	12 ± 2	103 ± 12	576 ± 12	Unstated	Unstated	[130]
		15	12 ± 2	44 ± 2	288 ± 12	720 ± 24	1 074 ± 40	
		20	12 ± 2	31 ± 1	216 ± 12	528 ± 24	787 ± 39	
		25	10 ± 2	22 ± 2	156 ± 12	336 ± 24	524 ± 40	
		30	8 ±1	12 ± 1	144 ± 12	288 ± 24	425 ± 38	
		35	8 ± 1	12 ± 1	144 ± 12	Unstated	Unstated	
2	*Sarcophaga argyrostoma* (Robineau-Desvoidy)	8	102*	215*	Unstated	Unstated	Unstated	[129]
		15	41*	43*	355*	879*	1 318*	
		20	24*	26*	245*	456*	751*	
		25	14*	16*	164*	339*	533*	
		30	12*	14*	125*	240*	391*	
		35	12*	12*	106*	228*	358*	
3	*Sarcophaga crassipalpis* Macquart	18	28.08*	42*	144*	501.12*	715.2*	[16]
		21	19.92*	34.08*	102*	312*	468*	
		24	18*	27.12*	108*	270.48*	423.6*	
		27	17.04*	18.96*	83.04*	216*	335.04*	
		30	14.4*	17.04*	75.6*	192*	299.04*	
		33	11.04*	12.48*	72*	168*	263.52*	
4	*Sarcophaga haemorrhoidalis* (Fallen)	15.6	14*	72*	186*	540*	812*	[128]
		21.1	12*	34*	114*	344*	504*	
		25.0	12*	32*	112*	300*	456*	
		26.7	6*	18*	86*	142*	252*	
		32.2	6*	18*	72*	264*	360*	
5	*Sarcophaga peregrina* (Robineau-Desvoidy)	16	56.0 ± 2.8	53.6 ± 2.2	170.0 ± 4.4	713.3 ± 30.0	1 064.7 ± 34.8	[131]
		19	40.5 ± 5.3	43.0 ± 2.0	121.3 ± 4.7	490.0 ± 16.2	756.0 ± 19.0	
		22	29.0 ± 1.0	28.6 ± 3.0	95.2 ± 1.8	366.8 ± 2.7	559.6 ± 5.5	
		25	20.3 ± 0.5	19.5 ± 1.0	70.0 ± 1.6	270.0 ± 5.2	414.3 ± 3.9	
		28	16.8 ± 1.8	15.6 ± 0.9	59.6 ± 2.2	200.6 ± 0.9	315.0 ± 2.0	
		31	14.5 ± 1.7	13.6 ± 2.2	53.5 ± 2.3	177.0 ± 1.7	278.0 ± 4.0	
		34	12.4 ± 0.9	12.2 ± 0.4	48.4 ± 3.0	170.0 ± 3.8	258.0 ± 3.5	
6	*Sarcophaga ruficornis* (Fabricius)	10	12 ± 2	120 ± 12	528 ± 12	Unstated	Unstated	[130]
		15	12 ± 2	24 ± 2	288 ± 12	768 ± 24	1 092 ± 40	
		20	12 ± 2	24 ± 2	156 ± 12	504 ± 48	696 ± 64	
		25	10 ± 2	12 ± 2	110 ± 12	288 ± 24	420 ± 40	
		30	4 ± 2	8 ± 2	108 ± 12	240 ± 24	360 ± 40	
		35	4 ± 2	8 ± 2	108 ± 12	240 ± 24	360 ± 40	
		16	Unstated	Unstated	Unstated	748.8 ± 26.6	1 166.4 ± 40	[127]
		19	Unstated	Unstated	Unstated	499.2 ± 18.2	751.2 ± 34.6	
		22	Unstated	Unstated	Unstated	434.4 ± 21.4	664.8 ± 28.6	
		25	Unstated	Unstated	Unstated	386.4 ± 15.6	592.8 ± 26	
		28	Unstated	Unstated	Unstated	273.6 ± 13.7	436.8 ± 15.4	
		31	Unstated	Unstated	Unstated	232.8 ± 14.2	381.6 ± 17.7	
		34	Unstated	Unstated	Unstated	225.6 ± 11.8	362.4 ± 15.8	
7	*Sarcophaga similis* Meade	15	52.0 ± 5.8	56.8 ± 7.5	161.2 ± 15.2	759.0 ± 16.8	1 029.0 ± 26.6	[132]
		17.5	33.3 ± 5.5	30.8 ± 5.6	136.0 ± 13.7	521.0 ± 12.6	731.0 ± 20.4	
		20	23.0 ± 3.7	26.0 ± 5.2	111.0 ± 16.1	408.5 ± 15.0	568.5 ± 20.8	
		22.5	19.3 ± 2.8	20.0 ± 4.8	94.0 ± 9.8	324.5 ± 9.0	457.8 ± 19.8	
		25	16.3 ± 2.5	14.0 ± 3.0	78.0 ± 7.0	239.3 ± 9.2	347.7 ± 14.6	
		27.5	16.0 ± 1.3	14.8 ± 3.5	64.0 ± 5.7	209.5 ± 7.4	304.5 ± 10.4	
		30	10.0 ± 1.0	9.7 ± 1.7	60.7 ± 5.0	186.7 ± 6.1	267.0 ± 9.2	
		32.5	11.3 ± 1.3	10.0 ± 1.4	55.0 ± 4.4	173.8 ± 6.0	250.0 ± 7.3	
		35	10.3 ± 1.5	8.0 ± 1.7	53.0 ± 6.2	166.0 ± 9.2	237.3 ± 7.7	

*Average stage duration.

In conclusion, the developmental duration of *S. ruficornis* from Central Arabian Peninsula is longer than that from south-eastern Brazil even at the same temperature [125,128]. At the constant temperature of 25 °C, the developmental duration of *S. ruficornis* is distinctly longer than that of *S. similis* [125,130]. Accordingly, the developmental durations of flesh flies should be related to the diversity of geography and climate in addition to the temperature and species. Therefore, further analysis of the developmental pattern of flesh flies at various temperatures in different geographic locations could improve the value of flesh flies in forensic investigations.
